# 

**Published:** 1902-07

**Authors:** 


### INDEX TO VOLUME XVIII.

Abortion Advertisements .................................. 24
Abstracts and Selections ...31, 145-151, 195-196, 295-300, 509-513
Actinomycosis .......................................... 235
A Doubting Thomas. Dr. L. L. Loggins, Wills, Texas........ 285
Address of W. H. Monday, M. D., to the Kaufman County Medical Society on His Retiring From the Presidency at Its Annual Meeting at Kaufman, January 13, 1903.......... 323
Adenoids and Hypertrophied Faucial Tonsils, as Portals of Infection—A Plea to the General Practitioner for their Early Recognition and Removal. By P. M. Payne, M. D., Brownwood, Texas ............................... 193
A Father in Medicine ..................................... 26
Alcohol. By E. E. Guinn, M. D., Rusk, Texas............... 491
Alcohol .................................................. 414
A Much Needed Hospital ................................... 29
American Medical Temperance Association ................. 430
American Medical Association ............................ 136
A Proposed Bill for a State Board of Health.............. 261
A Report of Cases of Abdominal Surgery. By Henry K. Leake, M. D., Dallas, Texas........................... 270
A Sheep-shearing Trust; John B. Murphy, M. D., LL. D., President ............................................ 501
A Step in the Right Direction............................. 75
A State Hospital for Consumptives Needed................. 115
A Study of Nature's Method for the Cure of Asiatic Cholera. By W. B. M'Laughlin, M. D., Austin, Texas.............. 412
A Timely Reminder ........................................ 74
Austin District Medical Society ......................... 239

Bearing Fruit ........................................... 144
Books and Magazines.36-40, 78-82, 120-126, 153-158, 244-246, 304-308, 343-354, 391-398, 442-444, 480-485, 516-518

Canine Rabies and a Case of Hydrophobia. By R. Menger, M. D., San Antonio, Texas ............................... 1
Care of the Perineum During and After Labor. By E. O. Boggs, M. D., Marquez, Texas ........................... 18
Cholera Infantum. By S. Burg, M. D., San Antonio, Texas. 98
Car and Hotel Disinfection .............................. 110
Charity .................................................. 287
Clinical Society of the New York Polyclinic Medical School and Hospital.............................................. 506

Delayed Union of Fractures, With Report of Three Cases. By J. C. Andrews, M. D., Granger, Texas................ 370
Death of Dr. Osborn .................................. 112
Dipththeria Antitoxin .................................. 114
Diluting and Washing to Blood in Certain Forms of Poisoning. By W. W. Greer, M. D., Cameron, Texas........... 500
Dr. Williams Arnold Adams .......................... 199
Dr. W. M. Vertrees .................................. 199
Dr. Frank Paschal, President State Medical Association.... 472

Easy on the Osteopath ................................ 75
Eclampsia. By W. H. Allen, M. D., Marlin, Texas..........489
Ergoapiol (Smith) in Diseases of the Female. By Charles H. Shepard, M. D., Physician to Lincoln Hospital, Dunham, N. C. ................................................ 62
Eye, Ear, Nose and Throat ..........330-332, 423-424, 449-450

Fire Proofing Essential in Modern Hospitals ............ 113

Infectious Endometritis. By J. W. Kennedy, M. D., San Antonio, Texas ...................................... 357

Johnson County Medical Society.......................... 424

Laboratory Bill for the Study of Criminal, Pauper and Defective Classes ...................................... 417
Ligation of the Dorsal Vein of the Penis as a Cure for Atonic Impotence. By Joe S. Wooten, B. S., M. D., Austin, Texas .................................................. 325
Love Amongst the Wise Men of the East................ 108

Madison County Medical Association .................. 468
Cases Reported. By J. M. Frazier, M. D., Belton, Texas.. 60
Meeting of the Tri-State Medical Society ................ 105
Membranous Stomatitis Complicating Pneumonia—Eight

Normal Salt Solution—When to Use It; How to Use It. By A. P. Heineck, M. D., Chicago ........................ 178
Neurasthenia in Relation to the Abdominal Diseases of Women. By Henry K. Leake, M. D., Dallas, Texas................ 401
No Bureau of Vital Statistics for Texas.................. 429
News and Miscellany ......32-35, 71-72, 116-120, 151-153, 239-244, 300-303, 332, 386-391, 436-442, 474-480, 513-516

Puerperal Eclampsia. By E. B. Parsons, M. D., Palestine, Texas .................................................. 366
Post-Graduate Medical School at Washington, D. C......... 234
Publisher's Notes ..40-48, 82-88, 126-128, 162-172, 214-216, 246-258, 308-312, 354-356, 398-400, 485-488, 520-526

Prophylaxis of Veneral Diseases ........................ 417
Recent Cases of Abnormal Surgery. By Henry K. Leake, M. D., Dallas, Texas ........................ 49
Report of a Case of Disarticulation of the Hip for Sarcoma of the Femur, With Remarks Upon the Diagnosis and Prognosis in Sarcoma of the Femur. By A. E. Halstead, M. D., Chicago ........................ 313
Report of a Case of Double Glioma Treated With X-Ray. By H. L. Hilgartner, M. D., Austin, Texas ........................ 322
Remarks on the Tractus Intestinalis By Byron Robinson, B. S., M. D., Chicago ........................ 363
Railway Car Sanitation Bill ........................ 431
Rheumatic Swellings Following Antitoxin Injections........ 236

Some Therapeutic Uses of Water. By L. M. Bassett, M. D., Hearne, Texas ........................ 4
Serum Therapy. By H. N. Graves, M. D., Georgetown, Texas ........................ 129
Social Aspects of Tuberculosis. By F. E. Daniel, M. D., Austin, Texas ........................ 217
Septicemia and the Curette. By H. Plymton, M. D., Brooklyn, N. Y. ........................ 328
Sterility in the Female, and Its Curability. By S. L. Kistler, M. D., Los Angeles, Cal ........................ 373
Sanitary Laws Enacted by the Twenty-eighth Legislature.... 432
Sabine County Medical Society ........................ 76
Smith County Medical Society ........................ 76
San Angelo District Medical Society........................ 202
State Medical Association of Texas, Thirty-fifth Annual Session, San Antonio, Texas........................ 450

Typhoid Fever and Its Treatment. By S. N. Aston, M. D., Jewett, Texas ........................ 14
The Use of Borax and Boric Acid as Food Preservatives...... 19
The Founding of a State Institution for Epileptics in Texas. By B. M. Worsham, M. D., Austin, Texas................ 89
Tinea Sycosis; With Report of Case. By Alexander A. Brown, M. D., San Antonio, Texas ........................ 134
The Drug Habit: Its Treatment. By M. K. Lott, M. D., Cameron, Texas ........................ 173
The Mosquito and Malaria. By A. N. Perkins, M. D., Sabine Pass, Texas........................ 231
The Rational Treatment of Snake-Bites and Others With Andrenalin Chloride. By R. Menger, M. D., San Antonio, Texas ........................ 445

The Public Health .................................... 21
The State Association's Problem of Problems.............. 26
The American Tuberculosis Congress ...................... 29
The Doctor's Self-inflicted Wrongs ...................... 73
The Easy Mark ........................................... 75
The Proposed State Board of Health ...................... 109
Texas to the Front ...................................... 111
The Governor's Appointments ............................. 139
The Painless Treatment of the Drug Habit................. 197
Transactions of the State Medical Association of Texas..... 199
The Public and the Doctor: Dr. Hadra's Book ............ 200
The State Board of Medical Examiners .................... 201
The West Texas Medical Society .......................... 201
The State Board of Health Bill .......................... 233
The Riot Act ............................................ 234
To the Texas State Medical Association and the Medical Profession of Texas ...................................... 291
The State Board of Health Matter ........................ 333
The Medical Practice Law Under Fire ..................... 377
The West Texas Medical Society Defeats Sanitary Legislation .................................................. 425
Texas Sets the Pace in Railway Car Sanitation—Dr. Johnson's Bill .............................................. 428
The San Antonio Meeting ................................. 434
The New Constitution .................................... 469
The Saratoga Meeting of the American Medical Association —Notes by the Wayside .............................. 66
The Reorganization Indifferences—Letter from Dr. Shropshire ................................................ 70
The Mosquito and Malaria ................................ 283
The Mandrake of the Bible ............................... 419
The East Texas Medico-Chirurgical Association ....105, 203, 468
The Southwestern Tri-State Medical Association.......... 136
Tri-State Medical Society of Alabama, Georgia and Tennessee. 136
The Brazos Valley Medical Association ............ ..138, 505
The South Texas Medical Association..................... 202
The Brazos Valley Medical Society—Announcement ........ 203
Treatment of Fracture in a Country Practice. By W. B. Collins, M. D., Lovelady, Texas........................ 497

West Texas Medical Association..........78, 106, 137, 201, 237
What a Doctor Should Do, and What He Should Not Do, in Case of Labor. By T. J. Bell, M. D., Tyler, Texas...... 224

